# Kir6.1- and SUR2-dependent K_ATP_ overactivity disrupts intestinal motility in murine models of Cantú syndrome

**DOI:** 10.1172/jci.insight.141443

**Published:** 2020-11-10

**Authors:** Nathaniel W. York, Helen Parker, Zili Xie, David Tyus, Maham Akbar Waheed, Zihan Yan, Dorothy K. Grange, Maria Sara Remedi, Sarah K. England, Hongzhen Hu, Colin G. Nichols

**Affiliations:** 1 Center for the Investigation of Membrane Excitability Diseases,; 2 Department of Cell Biology and Physiology,; 3 Center for the Study of Itch & Sensory Disorders, Department of Anesthesiology,; 4 Division of Endocrinology, Department of Medicine,; 5 Divison of Clinical Genetics, Department of Pediatrics, and; 6 Department of Obstetrics and Gynecology, Washington University School of Medicine, St. Louis, Missouri, USA.

**Keywords:** Gastroenterology, Muscle Biology, Genetic diseases, Muscle, Potassium channels

## Abstract

Cantú syndrome (CS), caused by gain-of-function (GOF) mutations in pore-forming (Kir6.1, *KCNJ8*) and accessory (SUR2, *ABCC9*) ATP-sensitive potassium (K_ATP_) channel subunit genes, is frequently accompanied by gastrointestinal (GI) dysmotility, and we describe 1 CS patient who required an implanted intestinal irrigation system for successful stooling. We used gene-modified mice to assess the underlying K_ATP_ channel subunits in gut smooth muscle and to model the consequences of altered K_ATP_ channels in CS gut. We show that Kir6.1/SUR2 subunits underlie smooth muscle K_ATP_ channels throughout the small intestine and colon. Knockin mice, carrying human *KCNJ8* and *ABCC9* CS mutations in the endogenous loci, exhibited reduced intrinsic contractility throughout the intestine, resulting in death when weaned onto solid food in the most severely affected animals. Death was avoided by weaning onto a liquid gel diet, implicating intestinal insufficiency and bowel impaction as the underlying cause, and GI transit was normalized by treatment with the K_ATP_ inhibitor glibenclamide. We thus define the molecular basis of intestinal K_ATP_ channel activity, the mechanism by which overactivity results in GI insufficiency, and a viable approach to therapy.

## Abstract 2

Smooth muscle Kir6.1/SUR2 channel overactivity reduces contractility and transit throughout the intestine, defining the molecular basis of gut KATP channels, the mechanism of GI insufficiency in Cantu syndrome, and a viable approach to therapy.

## Graphical abstract

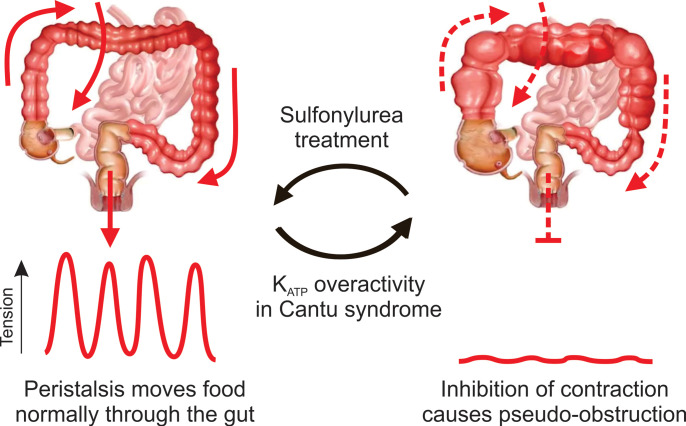

## Introduction

ATP-sensitive potassium (K_ATP_) channels, characterized by inhibition by intracellular ATP and activation by Mg-ADP, effectively link membrane excitability to metabolic state. Functional channels are composed of hetero-octameric complexes: 4 Kir6.x pore forming subunits, responsible for ATP inhibition, and 4 regulatory SURx subunits responsible for Mg-ADP activation. SUR subunits also impart sensitivity to agonists (diazoxide/pinacidil) and antagonists (glibenclamide) (1). Intriguingly there are 2 pairs of K_ATP_ genes, encoding SUR1 and Kir6.2 (*ABCC8, KCNJ11*) and SUR2 and Kir6.1 (*ABCC9, KCNJ8*), resulting in distinct subunit combinations in different cell types (2, 3), with additional structural heterogeneity provided by variable splicing in SUR1 and SUR2 (1, 4, 5). Kir6.2 and SUR1 are predominantly expressed in the pancreas and neurons, Kir6.2 and SUR2A in cardiomyocytes, and Kir6.1 and SUR2B in vascular smooth muscle (6–9). K_ATP_ channels are present in the gastrointestinal (GI) tract, but these have received less attention, and expressed isoforms are not entirely clear (10). Here, we demonstrate first that intestinal contractility is reliant on Kir6.1 and SUR2 expression in smooth muscle and, secondly, that pathogenic mutations within *ABCC9* and *KCNJ8*, both of which underlie Cantú syndrome (CS), result in impaired contractility and motility in both small intestine and colon.

After leaving the stomach, food moves down the length of the intestinal tract via segmented peristalsis, driven by slow waves produced in the pacemaking interstitial cells of Cajal (ICC). ICC exist in an electrically coupled syncytium together with smooth muscle cells (SMC) and PDGFRα^+^ cells, linking slow wave generation to SMC contraction (11). The organization of this process is coordinated by the enteric nervous system (ENS), allowing for reflexive control of movement patterns through modulation of Ca^2+^ release in ICC and SMCs (12, 13). All 4 K_ATP_ transcripts have been identified in ICC and, while both diazoxide and pinacidil have been shown to elicit current in ICC in culture, it appears that freshly isolated colonic ICC exhibit no pinacidil-sensitive current (14–16). Functional K_ATP_ channels are clearly present in intestinal SMCs, providing a target for K_ATP_ to influence contraction directly (11, 16). We are unaware of any definitive studies of subunit contributions to functional channels being carried out using gene KO animals, even though such studies have been carried out extensively in pancreas, nervous system, cardiac and skeletal muscle. In this study, we first used K_ATP_ subunit–KO mice, as well as tissue-specific knockdown of Kir6.1, to show that Kir6.1 and SUR2 are the major subunits underlying functionally relevant intestinal K_ATP_ channels in the SMCs.

Although these channels are not markedly active and do not influence contractility under basal conditions, contractility is dramatically inhibited by pharmacological activation. Patients with mutations in Kir6.1 or SUR2 that result in an increase in K_ATP_ channel function suffer from CS, characterized by congenital hypertrichosis, macrocephaly, coarse facial features, and complex cardiovascular features (17–20). Intestinal dysfunction is reported in a significant number of CS patients, generally appearing as severe constipation or slow intestinal motility, as well as gastroesophageal reflux and pyloric stenosis, with ~25% of patients requiring a feeding tube as infants or young children (21). Of the patients studied at the Washington University Cantú Research Clinic, one particularly dramatic example is a male subject with the common CS mutation SUR2[R1116H] (20, 21). This patient was born at 29 weeks gestation with significant edema, excessive scalp and body hair, and a patent ductus arteriosus that required surgery at 5 weeks of age; at 7 months of age, this patient was found to have an enlarged heart and small pericardial effusion, as well as pulmonary hypertension and a history of tracheobronchomalacia. As an infant, he was diagnosed with neuropathy of the GI system, including the small bowel and colon, requiring gastrostomy tube feedings. He was fitted with an antegrade continence enema (ACE) tube, through which he still receives nightly irrigation (1250 mL of saline with added bisacodyl and glycerin, infused into the colon through the ACE tube over 1 1/2 hours) at 14 years of age and, without which, he develops extreme constipation due to lack of intestinal motility.

Through analysis of mice in which CS mutations were introduced to the equivalent loci in the mouse *KCNJ8* and *ABCC9* genes, we previously demonstrated that vascular smooth muscle hypoexcitability underlies the cardiovascular features of CS (3, 22), but the multifaceted presentation in other tissues is not easily explained by chronic vascular dilation. Mice carrying more severe K_ATP_ gain-of-function (GOF) (homozygous Kir6.1[V65M], and double heterozygous Kir6.1[V65M]/SUR2[A478V]) CS mutations are associated with increased mortality, with death occurring around weaning (3). In these animals, we observe affected and distended bowels at the time of death, suggesting the possibility of GI dysfunction. We show here that even basal intestinal contractility and muscle tone are reduced in CS mice, in correlation with the molecular severity of the mutation. We further demonstrate that, in heterozygous Kir6.1[V65M] animals, reduced intestinal contractility results in significantly reduced intestinal transit rate, without disruption of intrinsic enteric neuronal activity. We thus identify K_ATP_ as a direct regulator of contractility via intestinal smooth muscle itself, providing a potential future target for treatment of CS symptoms, as well as of broader GI dysfunction.

## Results

### Kir6.1- and SUR2-dependent K_ATP_ channels can influence intestinal contractility.

**Figure 1:**
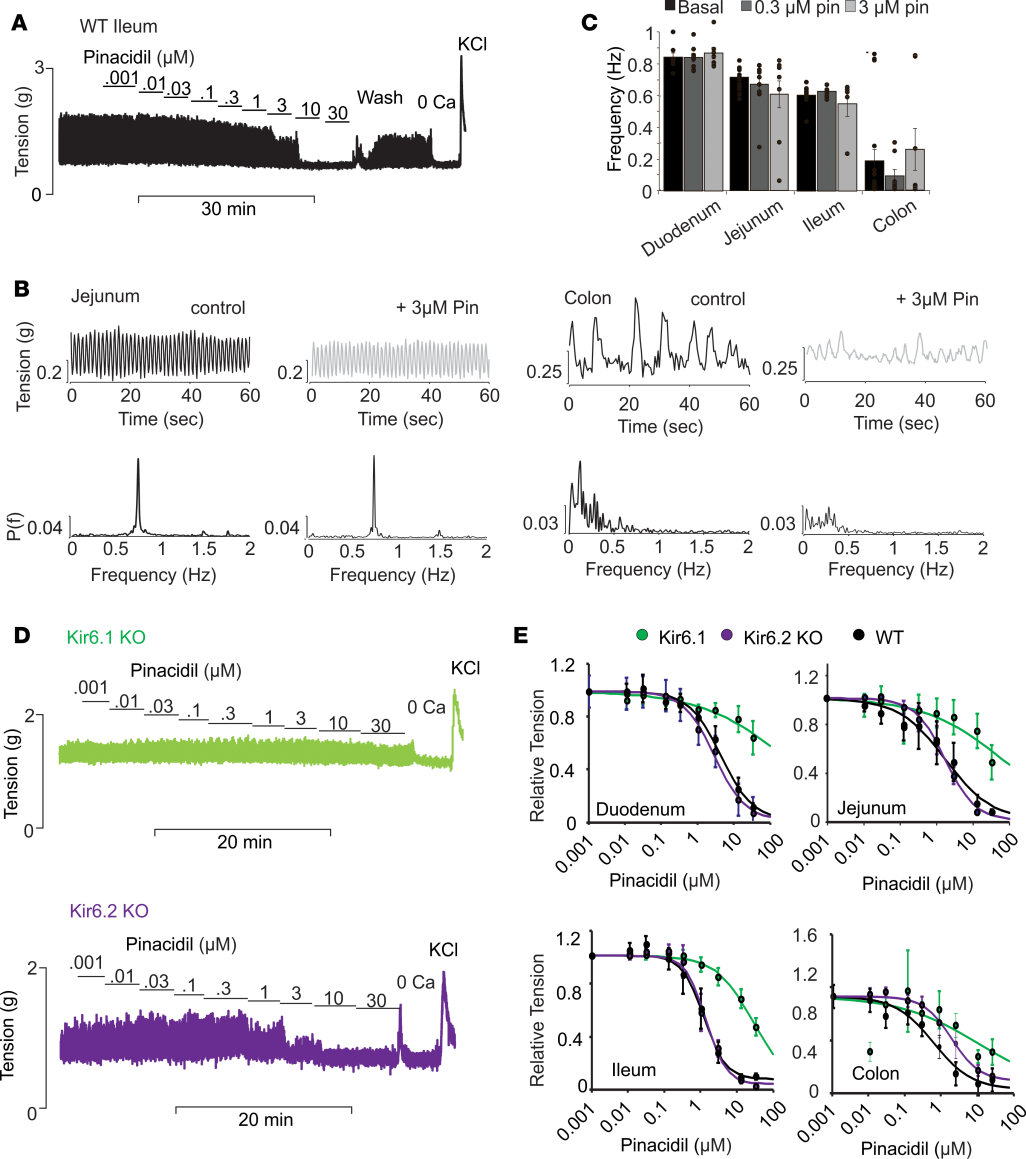
Kir6.1/SUR2 regulate intestinal contractility independently of pacemaker activity. (**A**) Representative recording of spontaneous contractions from WT ileum in the presence of increasing concentrations of pinacidil, followed by zero calcium (Ca) and high potassium (KCl). (**B**) Representative recordings of jejunal and colonic contractions in the absence and presence of pinacidil (above), and Fourier transform (below). (**C**) Dominant frequency of contraction at 0, 0.3, and 3 μM pinacidil in all segments, obtained from FFT analysis as in **B** (for duodenum, jejunum, ileum, and colon, *n* = 15, 14, 12, and 16, respectively). (**D**) Representative recordings of contraction in ileum segments from Kir6.1- and Kir6.2-KO mice following the same protocol as in **A**. (**E**) Pinacidil-response (relative tension) relationships for duodenum, jejunum, ileum, and colon from experiments as above (for WT duodenum, jejunum, ileum, and colon, *n* = 6, 5, 6, and 5, respectively. Kir6.1-KO duodenum, jejunum, ileum, and colon; *n* = 4, 5, 6, and 4, respectively. Kir6.2 -KO duodenum, jejunum, ileum, and colon; *n* = 5, 8, 4, and 7, respectively). Fits are arbitrary Hill plots for illustrative purposes only. In the KO animals, the pedestal may not be accurate.

To assess the role of K_ATP_ channels in control of intestinal contractility, we used combinations of pharmacology and genetic manipulation of K_ATP_ subunits, examining contractility in isolated intestinal segments (see Methods). Figure 1A shows a representative experiment on a segment from WT ileum. Similar recordings were obtained from duodenum, jejunum, and colon. In each case, contractions occurred rhythmically on top of a steady active tension (Figure 1B). Fourier analysis revealed a dominant frequency of 0.6–0.8 Hz in all segments, except the colon. Colonic contractions were less regular, and the dominant frequency was slower (Figure 1C).

To assess the consequence of SUR2-dependent K_ATP_ channel activation on contraction, the selective agonist pinacidil was added to the bath. Pinacidil caused a dose-dependent reduction in tension in all WT segments, with an IC_50_ of about 1–3 μM in all segments (Figure 1, A and E). Although Kir6.1 has long been presumed to be the relevant K_ATP_ channel pore-forming subunit, prior studies have demonstrated the presence of both Kir6.1 and Kir6.2 transcripts in intestinal muscle (10). We therefore also assessed pinacidil sensitivity in gut segments from Kir6.1- (23) and Kir6.2-KO mice (24). Kir6.1-KO segments exhibited a marked reduction in sensitivity to pinacidil in all segments, whereas pinacidil sensitivity was essentially unaltered by KO of Kir6.2, except in the colon (Figure 1, D and E). At 10 μM, pinacidil, WT duodenum, jejunum, ileum, and colon segment relative tension was reduced to 0.24 ± 0.09, 0.14 ± 0.07, 0.07 ± 0.02, and 0.12 ± 0.04, respectively, versus 0.79 ± 0.10, 0.73 ± 0.14, 0.45 ± 0.06, and 0.36 ± 0.07, respectively, in Kir6.1-KO segments, and versus 0.16 ± 0.07, 0.07 ± 0.02, 0.05 ± 0.03, and 0.26 ± 0.05, respectively, in Kir6.2 KO segments. These data confirm that Kir6.1 is the only relevant Kir6 subunit in mediating pinacidil-induced relaxation in the small intestine, although Kir6.2 subunits may also play a regulatory role in the colon.

### Functional Kir6.1 and SUR2 are present in intestinal smooth muscle.

**Figure 2:**
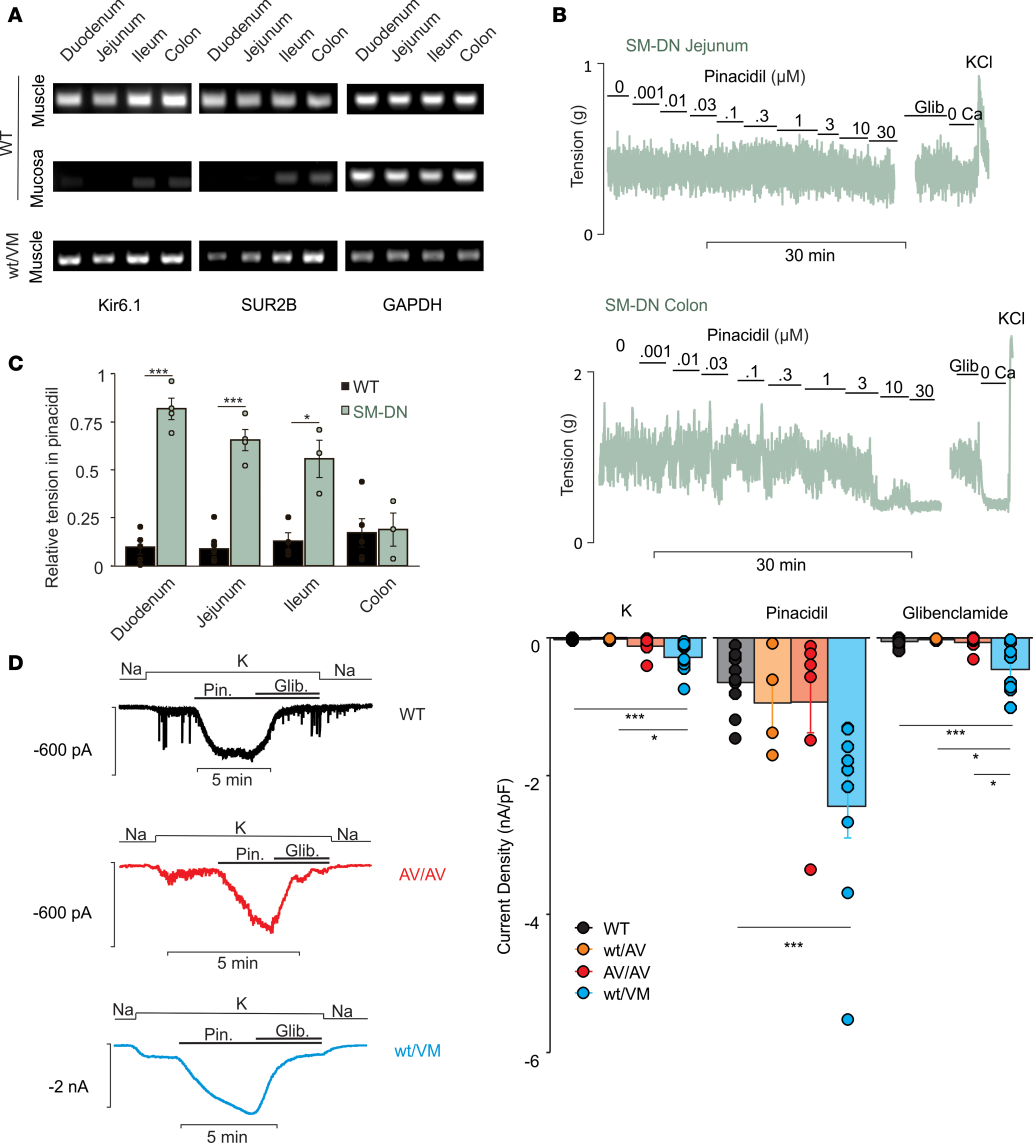
K_ATP_ action on intestine contractility is through smooth muscle. (**A**) RT-PCR shows expression of both Kir6.1 and SUR2B subunits specifically in the muscular layer of all segments of the GI tract. mRNA expression from WT/AV, AV/AV, and WT/VM were also assessed and showed consistent expression across all segments. WT/VM expression is shown here. (**B**) Representative recordings of spontaneous contractions in jejunum and colon from animals expressing a dominant-negative (Kir6.2[AAA]) construct specifically in smooth muscle (SM-DN), following the protocol shown in Figure 1A. (**C**) Relative tension in the presence of maximum (10 μM) pinacidil relative to the tension basal conditions (from experiments in **B** and Figure 1A). Significance was determined with Welch *t* test comparing SM-DN with WT for each segment. WT duodenum, jejunum, ileum, and colon; *n* = 5, 8, 4, and 5, respectively. SM-DN duodenum, jejunum, ileum, and colon; *n* = 4, 4, 3, and 3, respectively. (**D**) Representative whole-cell patch-clamp recordings and mean K^+^ current density (initial current in high Na^+^ solution subtracted), in isolated intestinal smooth muscle from WT and Cantú mice. Current density in high K^+^ solution is higher in SUR2^AV/AV^ and Kir6.1^WT/VM^, relative to WT, demonstrating GOF. Glibenclamide inhibited pinacidil-activated current in all genotypes, although inhibition was incomplete in Kir6.1^WT/VM^ (For WT *n* = 10 cells from 5 mice, SUR2^AV/WT^ *n* = 4 cells from 1 mouse, SUR2^AV/AV^ *n* = 7 cells from 2 mice, Kir6.1^WT/VM^ *n* = 9 cells from 2 mice). Significance was determined by 1-way ANOVA and post hoc Tukey’s test for pairwise comparison. Data shown as mean ± SEM (*P < 0.05, **P < 0.01, ***P < 0.001).

Previous analyses of K_ATP_ subunit expression have identified Kir6.1 and SUR2 in both intestinal SMCs and in the ICC (14–16). Importantly, the dominant frequency of contraction was unaffected by pinacidil; even as amplitude of contractions was reduced, the frequency was essentially unaffected (Figure 1, B and C). This suggests that the pinacidil action is on the SMCs, rather than the ICC. Consistent with this finding, we observed prominent mRNA expression of both Kir6.1 and SUR2B in intestinal smooth muscle but not the mucosa, throughout the small intestine and colon (Figure 2A).

Smooth muscle myosin heavy chain (SMMHC) is a ubiquitous marker of SMC lineage, and its promoter has been used to specifically target smooth muscle in many tissues, including the intestine (25, 26). We used mice expressing dominant-negative (DN) Kir6.1[AAA] subunits (SM-DN), under control of smooth muscle–specific SMMHC-Cre (27, 28), to specifically disrupt smooth muscle K_ATP_ channels (Figure 2, B and C). With the exception of the colon, this resulted in significant reduction of pinacidil sensitivity; duodenal, jejunal, and ileal segments were essentially insensitive to low (i.e., K_ATP_-specific) concentrations below 10 μM. Interestingly, while the pinacidil-insensitive component of WT contractility gradually increased along the intestines from proximal to distal, the pinacidil-insensitive component in SM-DN intestines gradually decreased to the same level, such that both WT and SM-DN colon exhibited similar levels of pinacidil insensitivity. This suggests that the potency of smooth muscle K_ATP_ activation gradually decreases from proximal to distal. We additionally confirmed the presence of K_ATP_ channels in intestinal SMCs through whole-cell patch-clamp recordings (Figure 2D) using a previously established protocol (14): in WT, small K currents were present under basal conditions at –70 mV, but large currents were induced by subsequent pinacidil addition, and these currents were inhibited by further addition of 10 μM glibenclamide. Together, these results demonstrate that, at least in the small intestine, the functionally relevant K_ATP_ channels are in the smooth muscle itself, but they suggest that this may not be the case in the colon.

### GOF CS mutations result in reduced basal contractility in the small intestine.

Having determined that Kir6.1/SUR2-dependent K_ATP_ channels are functionally relevant to intestinal contractility, we turned our attention to the potential pathological impacts of K_ATP_ GOF in CS. SUR2 and Kir6.1 mutations cause essentially the same CS features, and we previously generated 2 mouse models of CS, in which human CS mutations (A478V in SUR2 and V65M in Kir6.1) were knocked in to the mouse genome using CRISPR/Cas9 (3, 17). Both animals reiterate key cardiovascular features of CS, including low blood pressure and enlarged hearts (3, 22), but the impact on the GI system has not been assessed.

The effects of the introduced mutations on K_ATP_ channel function were determined using patch-clamp electrophysiology, as above. Basal K^+^ currents were markedly higher than WT in SUR2^AV/AV^, and more so in Kir6.1^WT/VM^ myocytes (Figure 2D). Maximum pinacidil-activated currents were also significantly higher than WT in Kir6.1^WT/VM^. Subsequent application of the K_ATP_ channel inhibitor glibenclamide markedly reduced the pinacidil-activated conductance in WT and SUR2^AV/AV^ myocytes, but it was less effective in Kir6.1^WT/VM^ (Figure 2D), potentially reflecting reduced glibenclamide sensitivity of Kir6.1[V65M] channels (29).

**Figure 3:**
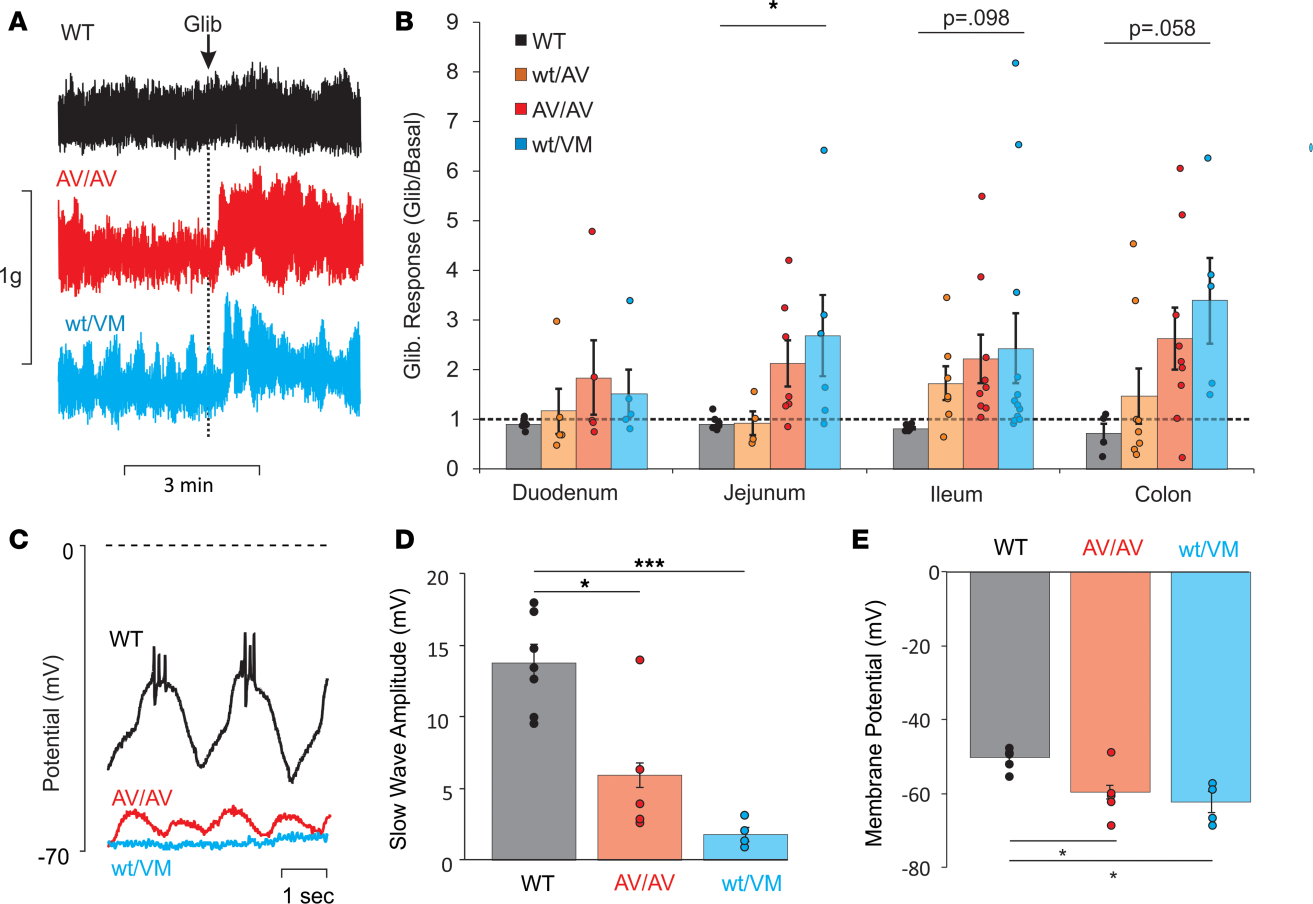
K_ATP_ GOF mutations in smooth muscle cause hyperpolarization and reduced basal contractility. (**A**) Representative records of basal tension in WT (black), SUR2^AV/AV^ (red), and Kir6.1^WT/VM^ (blue) ileal segments before (basal) and after application of glibenclamide (at arrow). (**B**) Tension in glibenclamide relative to basal, from experiments as in **A**. Significance was determined by 1-way ANOVA and post hoc Tukey’s test for pairwise comparison. WT duodenum, jejunum, ileum and colon; *n* = 6, 7, 7, and 4, respectively. SUR2^AV/WT^ duodenum, jejunum, ileum, and colon; *n* = 5, 4, 7, and 8, respectively. SUR2^AV/AV^ duodenum, jejunum, ileum, and colon; *n* = 4, 8, 8, and 9, respectively. Kir6.1^WT/VM^ duodenum, jejunum, ileum, and colon; *n* = 5, 8, 11, and 6, respectively. (**C**) Representative recordings of electrical activity in WT, SUR2^AV/AV^, and Kir6.1^WT/VM^ intestinal muscle segments, measured using impalement electrodes. (**D**) Slow wave amplitude measured from maximum hyperpolarized potential to maximum nonspiking plateau potential in WT, SUR2^AV/AV^, and Kir6.1^WT/VM^ muscle from experiments as in **A**. (**E**) Resting membrane potential (maximum hyperpolarized potential) in WT, SUR2^AV/AV^, and Kir6.1^WT/VM^ muscle. For **D** and **E**, *n* = 7 for WT, *n* = 4 for SUR2^AV/AV^, and *n* = for Kir6.1^WT/VM^. For each segment, significance was determined by 1-way ANOVA and post hoc Dunnett’s test, comparing each genotype with WT. Data are shown as mean ± SEM (*P < 0.05, ***P < 0.001).

Negligible basal K conductance in WT myocytes suggests that K_ATP_ activity will not normally play any prominent role under basal conditions. Accordingly, in WT intestinal segments, application of a high concentration of the K_ATP_ channel inhibitor glibenclamide (10 μM) had no obvious contraction-enhancing effects under basal conditions (Figure 3, A and B). In contrast, the much larger basal K^+^ conductance in CS myocytes could be expected to reduce even basal contractility. Indeed, glibenclamide caused a marked increase in both the pulsatile contraction amplitude and the steady tension in the jejunum, ileum, and colon of both heterozygous SUR2^WT/AV^, homozygous SUR2^AV/AV^, and heterozygous Kir6.1^WT/VM^ mice (Figure 3, A and B). The effect of glibenclamide increased with the expected severity of the molecular GOF (3) — from SUR2^WT/AV^ to SUR2^AV/AV^ to Kir6.1^WT/VM^ (Figure 3B).

These data demonstrate a decreased intrinsic basal contractility in CS small intestine and colon, due to increased basal K_ATP_ channel activity that can be inhibited by glibenclamide. Basal K_ATP_ conductance is expected to cause hyperpolarization of the muscle and suppression of the slow electrical waves that drive the elevation of intracellular [Ca^2+^] that underlies contractile activity. To assess this directly, we measured membrane potentials in intestinal segments using impaled sharp electrodes (Figure 3, C–E). In WT tissue, we observed slow waves with average amplitude of 13.7 ± 1.3 mV, and in mutant mice, there was a progressive decrease in amplitude in SUR2^AV/AV^ and Kir6.1^WT/VM^. In addition to this reduction in slow-wave amplitude, we observed an increase in the maximum hyperpolarization from about –50 mV in WT to about –65 mV in Kir6.1^WT/VM^ but with no obvious decrease in the frequency of oscillations (Figure 3; 0.50 ± 0.01 per second for WT, 0.43 ± 0.07 per second for SUR2^AV/AV^, 0.44 ± 0.07 per second for Kir6.1^WT/VM^). This is consistent with the consensus that slow waves are initiated in the ICC, which lack functional K_ATP_ (14), and are then conducted to and through the SMC syncytium.

### CS mutations reduce colonic contractility through direct inhibition of smooth muscle.

**Figure 4:**
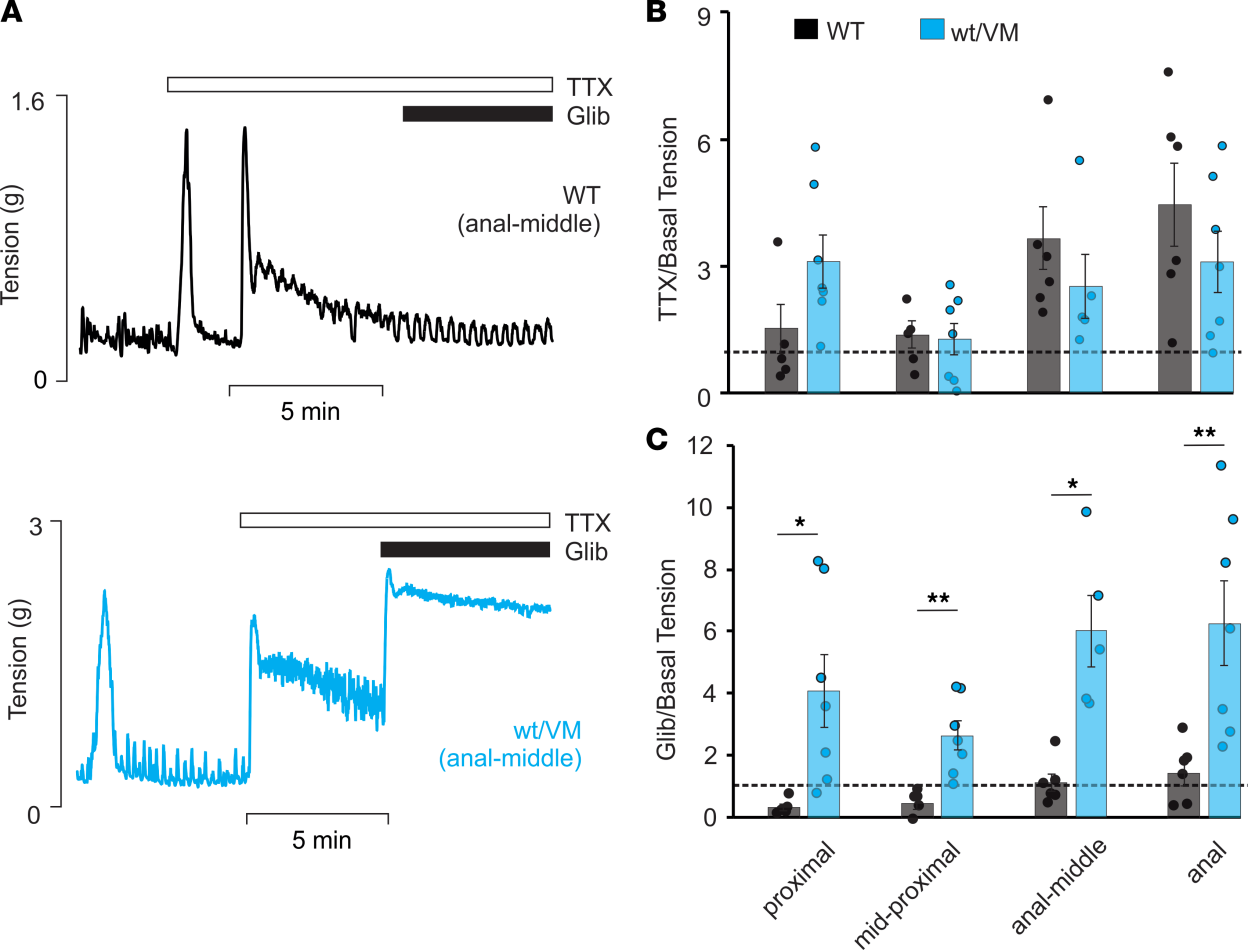
K_ATP_ GOF mutations in smooth muscle, not enteric neurons, reduce basal contractility. (**A**) Response to tetrodotoxin (TTX, 1 μM) followed by 1 μM glibenclamide in WT and Kir6.1^WT/VM^ colon segments. Abolition of neuronal activity with glibenclamide increased tension in both genotypes. Glibenclamide caused large increase in Kir6.1^WT/VM^ segments even after abolition of neuronal activity. (**B**) TTX induced contractions in all colonic segments of WT and Kir6.1^WT/VM^ segments. (**C**) Only Kir6.1^WT/VM^ segments exhibited a significant increase in tension in response to glibenclamide. WT, *n* = 6; Kir6.1^WT/VM^, *n* = 7. Each segment was compared with WT using Welch *t* test. Data shown as mean ± SEM (*P < 0.05, **P < 0.01).

A key component of normal GI transit is tonic suppressive control of colonic motility by the colonic migrating motor complex (CMMC), in turn controlled by the ENS (12, 30). Although neither Kir6.1 nor SUR2 is prominently expressed in neurons, this raises the possibility that reduced colonic contractility in CS tissues might be a consequence of altered neuronal control rather than a direct smooth muscle inhibition. By inhibition of neuronal Na^+^ channels, tetrodotoxin (TTX) suppresses enteric neurotransmission and increases colonic contractility (31). TTX (1 μM) increased tension similarly in both WT and Kir6.1^WT/VM^ colonic segments (Figure 4, A and B). Subsequent application of glibenclamide, in the continued presence of TTX, still causes increased tension in Kir6.1^WT/VM^, but not in WT, colonic segments (Figure 4, A and C). This indicates that the excitatory effect of glibenclamide on colonic contractility is present even when enteric neuronal activity is suppressed, further indicating that the effect is reliant on inhibition of basally active smooth muscle K_ATP_ in the CS colon.

### SUR2 and Kir6.1 mutants have different effects on pinacidil sensitivity.

**Figure 5:**
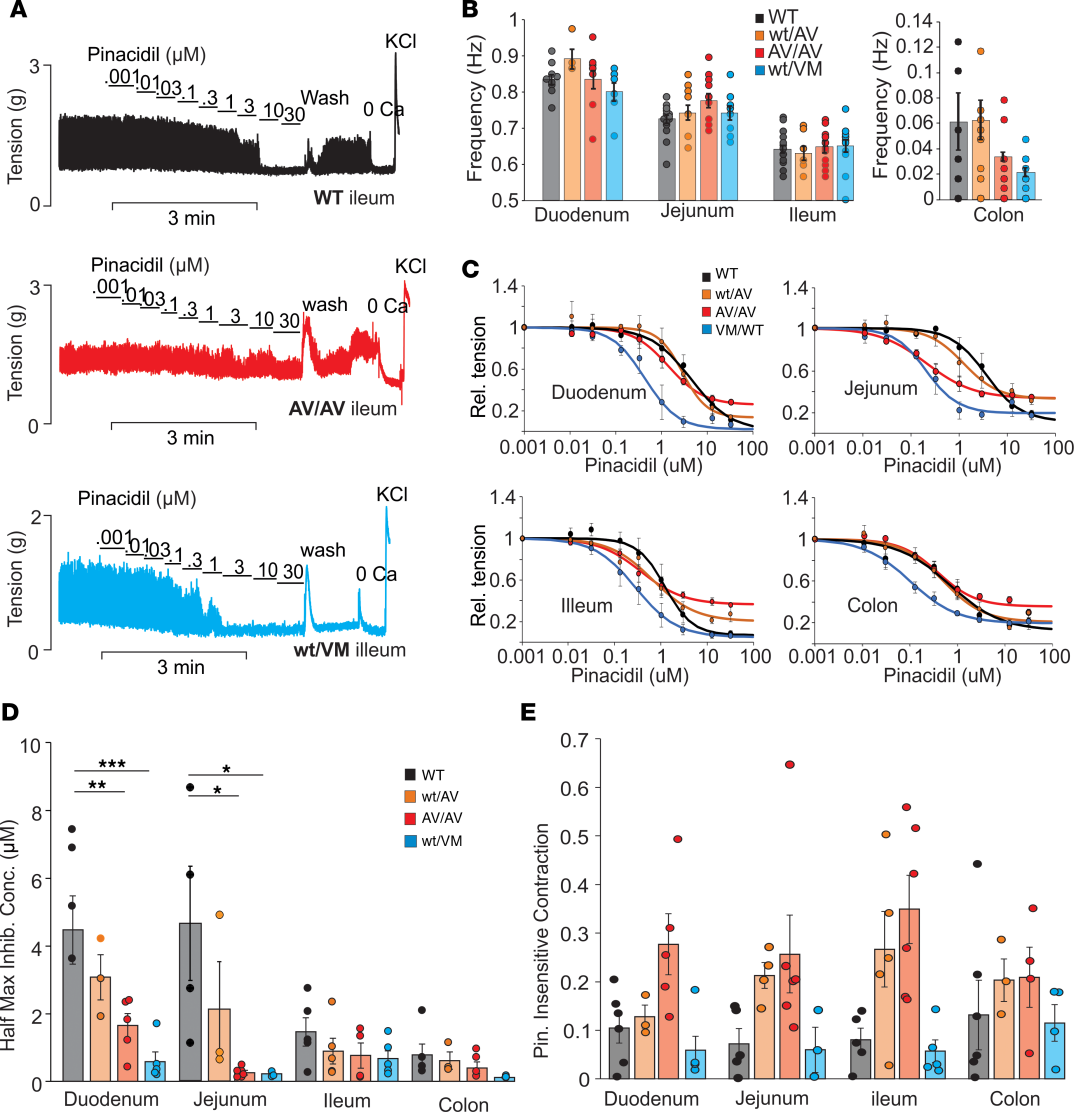
K_ATP_ GOF mutations do not affect intestinal contractile frequency but do alter pinacidil response. (**A**) Representative recordings of tension in WT (black), SUR2^AV/AV^ (red), and Kir6.1^WT/VM^ (blue) ileal segments in the absence and presence of pinacidil, following the protocol shown in Figure 1. (**B**) Fourier analysis reveals no significant effect of CS mutations on contractile frequency in any segments of the small intestine or the colon (WT duodenum, jejunum, ileum, and colon; *n* = 13, 17, 19, and 12, respectively. SUR2^AV/WT^ duodenum, jejunum, ileum, and colon; *n* = 4, 9, 7, and 10, respectively. SUR2^AV/AV^ duodenum, jejunum, ileum, and colon; *n* = 10, 12, 15, and 9, respectively. Kir6.1^WT/VM^ duodenum, jejunum, ileum, and colon; *n* = 10, 9, 13, and 11, respectively). (**C**) Pinacidil-response (relative tension) relationships for WT (black), SUR2^AV/AV^ (red), and Kir6.1^WT/VM^ (blue) duodenum, jejunum, and ileum, from experiments as above. SUR2^WT/AV^ and SUR2^AV/AV^ exhibit an increase in pinacidil-insensitive component of contraction, but Kir6.1^WT/VM^ do not. For illustrative purposes, average dose-responses are fit with single sigmoidal curves. (**D** and **E**) Half maximal inhibitory concentration in CS (**D**), and pinacidil-insensitive contraction as a fraction of the contraction in zero pinacidil (**E**), for SUR2^WT/AV^ (WT/AV) and SUR2^AV/AV^ (AV/AV) segments. For **C**–**E**, WT duodenum, jejunum, ileum, and colon; *n* = 6, 5, 5, and 5, respectively. SUR2^AV/WT^ duodenum, jejunum, ileum, and colon; *n* = 3, 3, 5, and 3, respectively. SUR2^AV/AV^ duodenum, jejunum, ileum, and colon; *n* = 5, 6, 4, and 4, respectively. Kir6.1^WT/VM^ duodenum, jejunum, ileum, and colon; *n* = 5, 3, 5, and 4 respectively). For all panels, significance was determined by 1-way ANOVA for each segment and post hoc Dunnett’s test, comparing each genotype with WT. Data are shown as mean ± SEM (*P < 0.05, **P < 0.01, ***P < 0.001).

While glibenclamide treatment demonstrates that all CS mutant intestinal segments have reduced basal contractility, mutant CS segments also show enhanced sensitivity to pinacidil. Reflecting a higher “activatability” of the mutant channels, this is more pronounced in SUR2^AV/AV^ versus SUR2^WT/AV^ segments and still more pronounced in Kir6.1^WT/VM^ segments, again paralleling the effect of the severity of the mutation on nucleotide sensitivity (3) (Figure 5, A–D). Interestingly, in SUR2^WT/VM^ and more so in SUR2^AV/AV^ segments, there was a marked pedestal of activity even at saturating pinacidil concentrations, an effect that was absent in Kir6.1^WT/VM^ segments (Figure 5E). Although further experiments would be necessary, this suggests the possibility that some compensatory effect, either a reduction in total SUR2 (and hence in K_ATP_ channel density) or perhaps an increase of an excitatory current, is present in the SUR2[A478V] tissues.

### CS V65M mutation markedly reduces GI transit in heterozygous animals and is lethal in homozygous animals.

**Figure 6:**
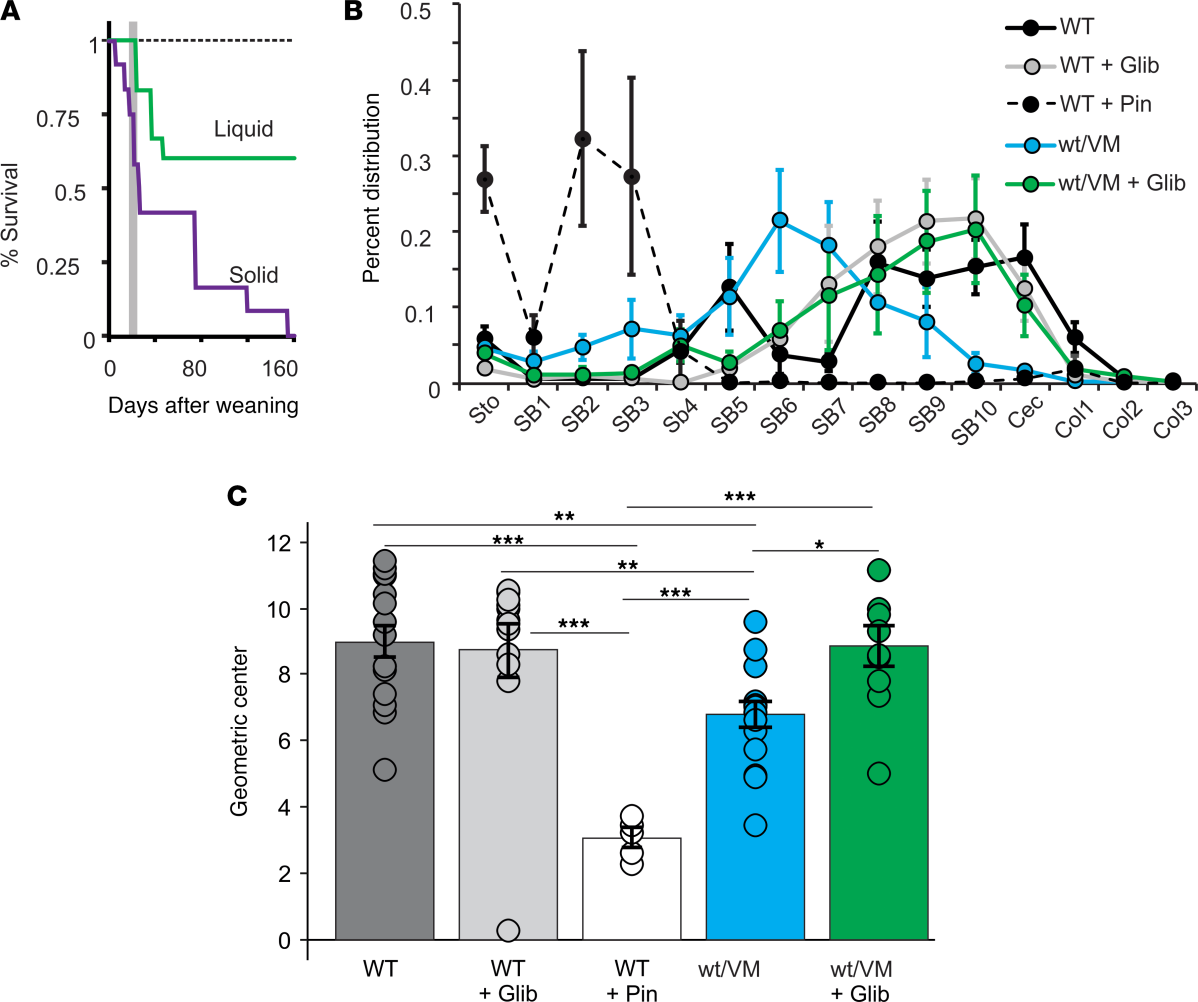
K_ATP_ GOF animals exhibit impaired transit, which can be corrected with glibenclamide. (**A**) Kaplan-Meier survival curves for homozygous Kir6.1^VM/VM^ animals when weaned onto solid food. There is significant mortality around the time of weaning onto solid food (gray line), but survival is improved when weaned onto liquid diet. (**B**) The distribution of FITC-dextran in equal length segments of the GI tract from stomach (Sto), through the small bowel (SB), the cecum (Cec), and colon (Col) at 90 minutes following oral gavage in WT and Kir6.1^WT/VM^ animals, with or without glibenclamide or pinacidil treatment. (**C**) The geometric center of FITC-dextran load from individual experiments as in **B**. For **B** and **C**, *n* = 15 for WT, *n* = 10 for WT + glibenclamide (Glib), *n* = 5 for WT + pinacidil (Pin), *n* = 15 for Kir6.1^WT/VM^, and *n* = 10 for Kir6.1^WT/VM^ + Glib. Significance was determined by 1-way ANOVA and post hoc Tukey’s test for pairwise comparison. Data shown as mean ± SEM (*P < 0.05, **P < 0.01, ***P < 0.001).

We previously reported that, although life expectancy was not markedly affected in heterozygous SUR2^WT/AV^ or Kir6.1^WT/VM^ animals, nor in homozygous SUR2^AV/AV^, more severely affected homozygous Kir6.1^VM/VM^ and double-heterozygous SUR2^WT/AV^/Kir6.1^WT/VM^ animals died around weaning (3), precluding further detailed study of phenotype. Anecdotally, at necropsy, we observe that Kir6.1^VM/VM^ intestines are typically filled with solid matter. Previous studies have recognized pseudo-obstruction of the intestines caused by loss of smooth muscle function (32–35), and we hypothesize that, in these animals, this is severe enough to preclude food transit through the gut following transition from milk to solid food. We have now attempted weaning homozygous V65M onto liquid gel diets (with the same nutritional composition as normal solid chow). While this does not completely abrogate the lethality, we do observe a striking increase in the number of Kir6.1^VM/VM^ mice surviving past weaning (Figure 6A), suggesting that inability to move solid food is indeed a direct cause of almost uniform lethality in the most severely affected CS mice.

Our isolated tissue data indicate a markedly reduced basal contractility in CS intestinal muscles (Figure 4). In vivo, this might be expected to reduce the rate of transit of food through the intestines. To test this directly, we administered a bolus of FITC–labeled dextran by oral gavage and determined the distribution through the gut 90 minutes later. Kir6.1^VM/WT^ mice exhibited significantly slowed transit, with the geometric center shifting from segment 10 in WT mice back to segment 7 in Kir6.1^VM/WT^ mice (Figure 6, B and C). To directly assess the impact of K_ATP_ activation on transit, we treated WT mice with a high dose of pinacidil (1 mg/mouse, i.p.) 30 minutes before dextran administration. Following pinacidil treatment, we observed an even greater slowing of transit, with the geometric center shifting to segment 3 (Figure 6, B and C).

### Pharmacological inhibition of overactive K_ATP_ can reverse transit defect in het V65M animals.

Finally, we sought to determine whether these transit deficits might be mitigated by pharmacological inhibition of K_ATP_ (22). We treated mice with high doses of glibenclamide (0.25 mg/mouse, i.p.), — 1 dose given 24 hours before assay and a second dose delivered immediately following FITC-dextran administration. Strikingly, glibenclamide had no significant effect on transit in WT animals but essentially completely normalized the geometric center in Kir6.1^WT/VM^ animals (Figure 6, A and B), demonstrating the potential of such an approach as treatment for GI dysmotility in CS.

## Discussion

### The role of K_ATP_ electrical regulation of GI motility.

GI motility arises from the coordinated activity of the intestinal SMCs, ICC, and PDGFRα^+^ cell (SIP) syncytium, the activity of which is in turn modulated by the ENS, allowing for the coordination of motility patterns. In this study, we have explored the role that K_ATP_ channels play in GI motility, confirming that K_ATP_ is present and functionally relevant in SMCs of the small intestine and colon, and demonstrating that GOF mutations in these channels can result in significant GI dysmotility.

The presence and identity of K_ATP_ channels in various cell types of the SIP syncytium have been reported previously, but their specific identity and the localization of relevant function has been unclear. Colonic SMCs have been shown to exhibit pinacidil- and ADP-activated currents, but ascribed to different Kir6 subunits in different studies (14, 36). While ICC in culture have been shown to express Kir6.1 and SUR2B in the colon but Kir6.2 and SUR2B in the small intestine, freshly isolated colonic ICC exhibit expression of both Kir6 isoforms but do not appear to have any K_ATP_ activity (14, 15). Consistent with them also having no relevant effect on ICC pacemaker activity, we show that activation of K_ATP_ by pinacidil dramatically reduces contractility of intestinal segments, but without altering the frequency of contraction. Previous studies have demonstrated that control of vascular tone by K_ATP_ is through Kir6.1/SUR2B-dependent channels in SMCs (3), and in this study, we confirm that these same channels are also acting through the smooth muscle in the intestine. Using gene KO animals, we first show that channels composed of Kir6.1, but not Kir6.2, are predominant in regulation of contractility throughout the intestines. This is demonstrated by dose-dependent pinacidil inhibition, which indicates involvement of SUR2 and which is significantly reduced in the absence of Kir6.1 but not Kir6.2. Our results are consistent with the recent observation that Kir6.1, but not Kir6.2, is expressed in colonic smooth muscle (14) and suggest that this may be the case throughout the intestinal tract. They also argue that smooth muscle is the functionally relevant location of K_ATP_ throughout. First, using whole-cell patch-clamp recordings in ileal SMCs, we show the presence of pinacidil-activated currents that are inhibited by glibenclamide, indicating the presence of functional K_ATP_ in these cells. Second, in mice expressing Kir6.1 or SUR2 GOF mutants, we observe a reduction in tone and reduced amplitude of contractions, but the frequency of contractions remains unaltered, suggesting that ICC pacing is unaffected. Finally, expression of DN Kir6.1(AAA) in smooth muscle (which will suppress both Kir6.2- and Kir6.1-dependent channels; ref. 25) significantly reduces the effect of pinacidil — to the level seen in Kir6.1-KO animals — throughout the small intestine. It is important to note that, even in the absence of Kir6.1/SUR2-dependent currents, there remains a low-affinity and incomplete sensitivity to pinacidil, an effect that is especially prominent in the colon. While the most likely explanation is a nonspecific drug action beyond surface K_ATP_ channels, it remains conceivable that there is a source of (presumably Kir6.2-dependent) K_ATP_ activity in tissues other than smooth muscle that nevertheless can have an effect on colonic contractility.

While most inputs from enteric neurotransmitters may be unlikely to be mediated by changes in the open probability of K_ATP_ channels, some bioactive mediators, including vasoactive intestinal peptide (VIP) (37, 38), pituitary adenylate cyclase activating polypeptide (PACAP) (39), and prostaglandins (38, 40), couple through Gs and enhanced cAMP, which can in turn activate K_ATP_. Thus, while our results argue that there is little effect of WT smooth muscle K_ATP_ on intestinal contractility under our basal experimental conditions, it is important to note that they do not preclude a role for humorally mediated K_ATP_ activation in normal postprandial motility or a role for an enhanced effect in CS.

### K_ATP_-dependent GI dysmotility in CS.

GOF mutations in either Kir6.1 or SUR2 underlie human CS (OMIM #239850) (41–43). While CS has principally been characterized by hypertrichosis, distinct facial appearance, cardiomegaly, and other cardiovascular features, GI dysfunction is also prominent in CS patients, with symptoms ranging from mild to very severe constipation (43). As we now report, in one particularly extreme example, the patient has been unable to defecate, resulting in years of total parenteral nutrition, followed by surgical implantation of an irrigation system, and even then requiring daily massage to achieve defecation. Mice carrying mutations analogous to CS-causing mutations in humans (Kir6.1[V65M] and SUR2[A478V]) reiterate the cardiomegaly and other vascular features of human CS (3), but we do not observe obvious GI problems, except in the more severely affected homozygous Kir6.1^VM/VM^ or double-heterozygous SUR2^WT/AV^/Kir6.1^WT/VM^ (3). As we now show, hetereozygous Kir6.1^WT/VM^ animals exhibit marked inhibition of intestinal transit, and homozygous Kir6.1^VM/VM^ die around weaning, unless weaned onto a liquid gel diet.

We also show that, in WT intestinal segments, basal contractility — at least under experimental conditions — reveals no obvious K_ATP_ channel activity. This is in contrast to vascular muscle, where we have good evidence for K_ATP_ channels playing a role even in basal tone in WT animals, such that K_ATP_ KO results in a hypertensive phenotype (7, 23, 44), and essentially all CS patients (21), as well as all CS mice (3), exhibit low vascular resistance and consequently enlarged hearts (22). While CS mice exhibit a clear increase in severity of both cardiovascular and GI pathology that correlates with the severity of the molecular defect (from SUR2[A478V] to Kir6.1[V65M] and from hetero- to homozygous presentation), GI problems in human CS are quite variable, and at this juncture, we cannot discern a clear molecular mutation disease severity correlation. One potential reason for a threshold effect of CS mutations on GI function (e.g., that some patients experience mild or no dysmotility, whereas others, such as we describe here, experience very severe problems — as is reiterated in the V65M CS mice) may depend on whether the mutational effect is sufficient to cross the threshold for reducing basal contractility and, hence, slowing down motility or not. Clearly, in some CS patients (and reiterated in mice with sufficiently marked mutagenic effect), the resultant GI dysmotility is severe, and a therapeutic remedy is warranted. Glibenclamide or other inhibitors of K_ATP_ channels show promise as therapeutic agents to directly treat CS pathology in the cardiovascular system (22, 45). As we show here, acute treatment with glibenclamide also causes striking reversal of the transit deficiency in Kir6.1^WT/VM^ animals, dramatically illustrating the potential for glibenclamide treatment of CS GI dysmotility and raising the further possibility that such treatments might be effective in any other GI dysmotilities resulting from decreased smooth muscle excitability. In principle, since Kir6.1/SUR2B-dependent channel activity will be enhanced in all relevant tissues, glibenclamide or another inhibitor might be expected to improve outcome in all such tissues in CS. However, it is worth noting that SUR2 is also implicated in the myocardial transition from glycolytic to oxidative metabolism that occurs during adaptation to postnatal life (46) and that SUR2-deleted cardiomyocytes exhibit enhanced glucose uptake (47), suggesting caution regarding potential metabolic consequences of SUR2 inhibition. Moreover, as has been discussed extensively elsewhere (48–52), none of the available K_ATP_ channel inhibitors are specific for these isoforms, and actions on Kir6.2/SUR1-dependent channels could lead to serious complications. Potentially the most dangerous unwonted effect is lowering of blood sugar levels as a result of action on pancreatic Kir6.2/SUR1-dependent channels. While this does not appear to be a significant issue in CS mice (22), this may not be the case in human CS, and there remains a need for subunit-specific agents.

### Conclusions.

Kir6.1- and SUR2-dependent K_ATP_ channels are expressed and functional in intestinal smooth muscle, and they can influence contractility throughout the intestine. Mutations associated with CS, which result in a GOF of these K_ATP_ channels, reduce smooth muscle contractility throughout the intestinal tract. This reduced contractility is sufficient to disrupt intestinal transit, and in the most severe cases, it can cause significant morbidity, explaining GI problems in CS patients (3). The K_ATP_ inhibitor glibenclamide, which reverses key cardiovascular features of CS, can also restore GI transit rate in experimental CS. This suggests the exciting and obvious potential for their use to offer relief from the GI symptoms of CS, and possibly from other GI dysmotilities that may result from decreased smooth muscle excitability.

## Methods

### Mouse models.

Kir6.1-KO mice, generated by deletion of part of intron 2 and exon 3 of the *Kcnj8* gene that includes the pore-forming region of the channel (23), were a gift from Susumu Seino (Chiba University, Chiba, Japan). DN Kir6.1[AAA] was expressed in an inducible, tissue-specific manner by crossing Tg[CX1-EGFP-Kir6.1(AAA)] (Kir6.1[AAA]) mice (25) (a gift from William Coetzee, New York University, New York, New York, USA) with transgenic mice (SM-Cre) that express inducible Cre recombinase (CreER^T2^) driven by the SMMHC promoter (53). Eight-week-old mice were treated with serial tamoxifen injections (1 injection of 50 μg/g body weight per day for 5 days) to induce DN Kir6.1[AAA] expression, and experiments were performed 4 weeks after induction. Generation of Cantú mice using CRISPR/Cas9 genome editing was previously reported (3). Briefly, *KCNJ8* (c.193G>A/195A>G) and *ABCC9* (c.1427C>T) mutations were introduced to mimic human CS Kir6.1[V65M] and SUR2[A487V] mutations, respectively. Heterozygous Kir6.1[V65M] (Kir6.1^WT/VM^) and SUR2[A478V] (SUR2^WT/AV^) mice mimic the autosomal-dominant context observed in patients. SUR2^WT/AV^ mice and Kir6.1^WT/VM^ mice were each in-crossed to generate homozygous mutant mice (SUR2^AV/AV^). Male and female mice were used for all experimental groups. Littermate WT mice were used for all comparisons.

### Isometric tension recordings.

Adult male and female mice were euthanized by overdose of 2.5% Avertin followed by cervical dislocation. Laparotomy was then performed, and the whole intestine was excised and placed into ice cold Krebs-Ringer solution containing (in mM) NaCl 120, KCl 5.9, CaCl_2_ dihydrate 2.5, MgSO_4_ 1.2, NaH_2_PO_4_ 1.2, NaHCO_3_ 15.5, and glucose 11.5. The pH of the solution was adjusted to 7.4 using HCl/NaOH. All chemicals were obtained from Sigma-Aldrich. A total of 1–2 cm intestinal segments was cut from the duodenum, jejunum, ileum, and colon. Adipose and mesentery tissue were carefully removed using micro scissors, and feces were flushed out using Krebs-Ringer solution. Intestinal segments were tied to metal brackets on both ends using 4-0 silk surgical suture and mounted in individual organ baths containing 25 mL Krebs-Ringer solution bubbled with 95% O_2_/5% CO_2_ at 37°C, to a force transducer for isometric tension recording. Tension was adjusted to ~1.0 g. Baseline tone was then typically relaxed to a stable ~0.5 g, and contraction amplitude was increased to a stable level, over a stabilization period of 15–45 minutes. Unless otherwise noted, the standard experimental protocol involved addition of pinacidil (0.01–30 μM) by serial additions to the bath solution at 4–5 minute intervals. The bath solution was then twice replaced with modified Krebs-Ringer solution without calcium (in mM) NaCl 120, KCl 5.9, MgSO_4_ 1.2, NaH2PO_4_ 1.2, NaHCO_3_ 15.5, glucose 11.5, EGTA 4. The intestinal segments were left to recover in the modified zero Ca Krebs-Ringer solution for 15–20 minutes to achieve minimal tension. Maximal tension was then assessed by replacement of bath solution with a modified high [K^+^] Krebs-Ringer solution (in mM) KCL 126, CaCl_2_ 2.5, MgS0_4_ 1.2, NaH_2_PO_4_ 1.2, NaHCO_3_ 15.5, glucose 11.5. Tension in each condition was calculated by averaging the tension during the final 1 minute before the following treatment, with the exception of the high K response, for which the maximum value was taken. Relative tension is defined by the following equation:

Relative tension = (tension – tension in 0 Ca)/(maximal tension in high K – tension in 0 Ca).

Tension recording was obtained using AdInstruments Octal Bridge Amp, PowerLab 8/30, and LabChart 7 and recorded at a 20 Hz.

Contractile frequency was assessed by fast Fourier transform (FFT) of the tension signal during the final 1 minute in control and pinacidil conditions, using the Fourier analysis tool in Microsoft Excel.

### Intestinal transit times.

Intestinal transit was determined as in previously published studies (54, 55). A total of 200 μL of a 2.5 mg/mL nonabsorbable 70 kDa fluorescein isothiocyanate – labeled dextran (FD70) solution — was administered by oral gavage. After 90 minutes, the animals were anesthetized with isoflourane and sacrificed. The GI tract was removed and divided into 15 segments (the stomach, 10 equal segments of the small intestine, the caecum, and 3 equal sized segments of the colon). The intraluminal contents were flushed out of each segment with 1 mL of PBS and collected. The samples were centrifuged, and the supernatant was assessed fluorometrically for concentration of FD70. Transit was determined by calculation of the geometric center of the distribution of FD70 (Ʃ for concentration signal in each segment × segment number)/100).

Colonic transit was determined using a glass bead expulsion assay. A glass bead (3 mm) was inserted 2 cm into the colon of a mouse after 12 hours of fasting. Expulsion time was measured for each mouse, and the average for each genotype was reported.

### Microelectrode recordings.

Intestinal segments were isolated as described above. Intact segments were stretched and pinned to the sylgard base of a tissue bath to prevent excessive movement and were perfused with Krebs-Ringer solution, maintained at 37°C, and continuously bubbled with 95% O_2_/5%CO_2_. The tissue was impaled with thin glass microelectrodes of 10–20 MΩ resistance (when filled with 3 mM KCl), and the membrane potential was digitized by a Power 1401 A/D converter and recorded via PClamp. Recordings were taken when a stable resting membrane potential or stable oscillating potentials were observed. Reported membrane potential was determined by averaging minimum potential in 3–5 slow waves, and amplitude — measured from trough to peak — was averaged across multiple slow waves.

### Patch-clamp electrophysiology.

Adult mice were anesthetized with 2.5% avertin (10 mL/kg, i.p.; MilliporeSigma) and cervical dislocation was performed. The whole intestine was removed, from duodenum to rectum. Extraneous tissue was removed, and the small ileal segment was removed, segmented, and placed in M199 cell media (1× M199, 26 mM NaHCoO_3_, 2 mM glutamine, 1% penicillin-streptomycin, 10% FBS). The tunica muscularis was peeled away along the deep muscular plexus margin, cut into small pieces, and placed in dissociation solution A (134 mM NaCl, 3 mM KCl, 5 mM Taurine, 5 mM EDTA, 2 mM MgCl_2_, 10 mM HEPES). Tissue was then spun down, the supernatant was removed, and dissociation solution A with 1 mg/mL trypsin was added, followed by 15 minute incubation at 37°C. After 15 minutes, this solution was replaced with fresh dissociation solution A plus 1 mg/mL trypsin and incubated for further 30 minutes at 37°C. The tissue was briefly centrifuged, and the supernatant was removed and replaced by dissociation solution B (134 mM NaCl, containing 3 mg/mL collagenase and 1 mg/mL BSA) and then further incubated for 33 minutes at 37°C. After incubation, the mixture was shaken at 800 rpm for 2 minutes, and the cell suspension was removed with effort made to limit the intact debris, layered on top of 20% (w/v) Ficoll, and centrifuged at 20*g* for 15 minutes at room temperature. The cell band located at the interface was removed, and M199 media was added. Cells were resuspended in fresh M199 and plated on glass coverslips for recording.

Whole-cell K_ATP_ currents were recorded using an Axopatch 200B amplifier and Digidata 1200 (Molecular Devices), following a previously published protocol for characterization of Kir6.1/SUR2 in SM cells (3). Recordings were sampled at 3 kHz and filtered at 1 kHz. Currents were initially measured at a holding potential of –70 mV in a high-Na^+^ bath solution containing (in mM) 120 NaCl, 6 KCl, 2.5 CaCl_2_, 1.2 MgCl_2_, 10 HEPES, and 12 glucose, with pH adjusted to 7.4 with NaOH before switching to a high-K^+^ bath solution (130 KCl, 2.5 CaCl_2_, 1.2 MgCl_2_, 10 HEPES, and 12 glucose, with pH adjusted to 7.4 with KOH) in the absence and presence of pinacidil and glibenclamide as indicated. The pipette solution contained (in mM) 110 potassium aspartate, 30 KCl, 10 NaCl, 1 MgCl_2_, 10 HEPES, 0.5 CaCl_2_, 4 K_2_HPO_4_, and 5 EGTA, with pH adjusted to 7.2 with KOH.

### Statistics.

Statistical significance was determined using the 2-way Welch *t* test or 1-way ANOVA followed by post hoc Tukey’s test or Dunnett’s test, as appropriate, and indicated in figure legends. Welch test was performed using Microsoft Excel. ANOVA and Tukey’s test were performed using R. Dunnett’s test was performed using DescTools package in R. Unless otherwise noted, data are presented as mean ± SEM. *P* < 0.05 was considered significance and P values were denoted as *P < 0.05, **P < 0.01, ***P < 0.001).

### Study approval.

All studies were performed in compliance with the standards for the care and use of animal subjects defined in *Guide for the Care and Use of Laboratory Animals* (National Academies Press, 2011) and were reviewed and approved by the Washington University IACUC.

## Author contributions

NWY, HP, MSR, HH, and CGN conceived the study; NWY, HP, ZX, DT, MAW, and ZY carried out the experiments; DKG, SKE, MSR, and HH contributed key technical help; NWY and CGN wrote the paper, which was edited by the other authors.

## Version 1 11/10/2020

In-Press Preview

## Version 2 12/03/2020

Electronic publication
